# Efficacy and Safety of Sustained-Release Melatonin Capsules (2 mg) in Healthy Adults with Poor Sleep Quality: A Randomized, Double-Blind, Placebo-Controlled Trial

**DOI:** 10.3390/clockssleep8020031

**Published:** 2026-05-27

**Authors:** Shefali Thanawala, Rajat Shah, Alphy Lopes, Milind Kulkarni, Bharat Jain, Niranjan Andhalkar

**Affiliations:** 1 Nutriventia Private Limited, Mumbai 400069, Maharashtra, India; 2 Kulkarni Clinic, Pune 411015, Maharashtra, India; 3 Dhanwantari Hospital, Pune 411002, Maharashtra, India; 4 ProRelix Services LLP, Pune 411052, Maharashtra, India

**Keywords:** polysomnography, PSQI, sleep efficiency, sleep onset latency, sleep quality, sustained-release

## Abstract

Sleep disturbances and poor sleep quality are growing public health concerns, adversely affecting both physical and mental health. While exogenous melatonin supplements are used to manage the condition, there is limited evidence available on the efficacy of sustained-release (SR) melatonin formulations. This multicenter, randomized, double-blind, placebo-controlled clinical trial evaluated the efficacy and safety of melatonin-SR capsules (2 mg) in healthy adults with poor sleep quality. Participants aged 30–60 years with poor sleep quality received melatonin-SR (2 mg) or a placebo capsule at night for 28 days. Changes from baseline to day 28 in polysomnography (PSG)-derived sleep parameters, Pittsburgh Sleep Quality Index (PSQI), WHO-5 Well-Being Index, sleep diary parameters, and safety profile were evaluated. Of 62 enrolled participants, 59 (melatonin-SR, *n* = 28; placebo, *n* = 31) completed the study. Compared with placebo, melatonin-SR supplementation resulted in significant improvements at day 28 in PSG-derived sleep efficiency (change from baseline: 3.49 for melatonin-SR vs. −6.30% for placebo; *p* = 0.001) and total sleep time (change from baseline: 23.83 for melatonin-SR vs. −39.25 min for placebo; *p* = 0.001), along with significant reductions in sleep onset latency (change from baseline: −10.28 for melatonin-SR vs. 16.70 min for placebo; *p* = 0.031) and wake after sleep onset (change from baseline: −14.92 for melatonin-SR vs. 24.71 min for placebo; *p* = 0.001). Melatonin-SR supplementation demonstrated a large treatment effect for the improvement in sleep efficiency compared with placebo (Cohen’s *d* = 0.9). A significant reduction in PSQI global scores was observed in the melatonin-SR group from day 07 onwards (change from baseline on day 07: −2.21 vs. −0.23; day 14: −4.86 vs. −0.65; and day 28: −5.61 vs. −0.65 for melatonin-SR and placebo, respectively; *p* = 0.001). Improvement in subjective psychological well-being was significant from day 14 onwards (change from baseline on day 14: 9.86 vs. 0.77; and day 28: 13.29 vs. 0.77 for melatonin-SR and placebo, respectively; *p* = 0.001). A significant improvement in subjective sleep parameters at day 28 (*p* < 0.05) was observed. Reported adverse events in both groups were mild and transient in nature. Supplementation with melatonin-SR 2 mg capsule at night for 28 days was found to be effective and safe in improving objective and subjective sleep quality outcomes and overall well-being in the trial population.

## 1. Introduction

Healthy sleep, characterized by multiple dimensions including sleep duration, efficiency, timing, alertness, and quality, is a vital parameter responsible for maintaining health throughout the human life span, as it profoundly influences physical, cognitive, and emotional well-being [1,2]. Epidemiological data indicate that around one in three (estimated 83.6 million) adults in the United States do not achieve adequate sleep on a regular basis [3]. According to the National Health Interview Survey (NHIS) conducted in the United States, nearly 15% of adults reported difficulty in initiating sleep and 18% reported problems with sleep maintenance. A similar trend of insufficient sleep has been reported in other parts of the world as well [4,5]. Evidence from a recent meta-analysis of sleep disorders indicates that insomnia affects approximately 25% of the Indian population, and nearly 15% of otherwise healthy individuals report sleep-related problems such as insomnia and obstructive sleep apnea, highlighting sleep disturbances as an important and potentially underrecognized public health concern in the country [6].

Irrespective of the causative factor, sleep disturbances are widely recognized to adversely affect both physical and mental health. Poor sleep quality, characterized by prolonged sleep latency and frequent nocturnal awakenings, is associated with increased fatigue and has been linked to a broad range of adverse health outcomes, including impairments in attention, memory, and learning, as well as an increased risk of mood disorders (such as anxiety and depression) and metabolic disorders (including obesity and diabetes) [5]. Given the close relationship between disrupted sleep and these conditions, impaired sleep has a substantial adverse impact on the overall quality of life [7]. The American Academy of Sleep Medicine (AASM) and the Centers for Disease Control and Prevention (CDC) have identified insufficient sleep and the resulting sleep debt as significant ‘public health concerns’, underscoring the need for effective and sustainable management strategies [2,3,8].

Several pharmacotherapy options, including benzodiazepine receptor agonists, benzodiazepines, or melatonin receptor antagonists, are available for the management of sleep health issues; however, the clinical utility of benzodiazepine and benzodiazepine receptor agonists is often limited by safety and tolerability concerns, especially when aimed at long-term use. Commonly reported adverse effects of these pharmaceutical agents include excessive daytime somnolence, cognitive impairment, poor tolerability, and the potential for dependence and withdrawal symptoms, which may compromise long-term adherence to these products [9,10].

Melatonin (N-acetyl-5-methoxytryptamine), an endogenous chronobiotic hormone produced by the pineal gland, plays a central role in synchronizing and promoting the normal sleep-wake cycle by regulating circadian rhythms. Melatonin secretion follows a circadian pattern, with levels rising in the evening to promote sleep onset and declining toward morning [11]. Disruptions in circadian rhythms or alterations in melatonin secretion due to aging, lifestyle factors, stress, or increased exposure to artificial light have been implicated in sleep disturbances, particularly those involving delayed sleep onset and fragmented sleep [12,13,14]. In line with the crucial role of endogenous melatonin in the maintenance of healthy sleep, supplementation with exogenous melatonin is being widely investigated and utilized across the globe for the management of sleep-related complaints, including insomnia and circadian rhythm sleep–wake disorders, as well as sleep disturbances associated with jet lag and night-shift work [15].

While conventional immediate-release melatonin formulations are effective in facilitating sleep onset, their short plasma half-life limits their ability to sustain sleep throughout the night. Consequently, such formulations may have limited impact on sleep maintenance and nocturnal awakenings, underscoring the need for alternative delivery systems that provide prolonged melatonin exposure. To address these limitations, novel sustained-release formulations of melatonin that can mimic the physiological nocturnal secretion profile have been developed. These formulations are designed to release an initial proportion of the active compound (up to 50%) shortly after administration, followed by a gradual and continuous release over an extended period. This release pattern helps maintain more stable plasma melatonin levels throughout the night and minimizes the peak-and-trough fluctuations commonly associated with immediate-release melatonin products [16,17]. Therefore, these formulations may offer advantages in improving sleep continuity, sleep efficiency, and overall sleep architecture. Melotime^™^ (Nutriventia Private Limited, Mumbai, India) is a sustained-release melatonin formulation (Melatonin-SR) developed to provide uniform release and maintenance of melatonin levels in the plasma throughout the 8 h sleep period, followed by gradually tapering plasma levels, thus avoiding any spillover of sleep during the waking hours. A comparative pharmacokinetic study between melatonin-SR and an immediate-release melatonin reference product after single-dose oral administration in healthy adults demonstrated that melatonin-SR notably maintained sustained plasma melatonin concentrations for an extended period, which can help induce and maintain sound sleep for long hours in healthy adults [17].

Despite the growing commercial availability of melatonin supplements, evidence supporting the efficacy and safety of these in achieving optimal sleep health from well-designed randomized, placebo-controlled trials, particularly those employing objective sleep assessments, remains limited. Polysomnography (PSG), the gold-standard method for objectively evaluating sleep architecture and physiological parameters during sleep [18], when combined with validated subjective sleep measures, can offer a comprehensive approach for assessing the clinical efficacy of sleep interventions. However, the PSG-based evaluations of sustained-release melatonin supplements have been limited and largely confined to populations with diagnosed sleep disorders, with scarce data in otherwise healthy adults experiencing poor sleep quality [19,20]. To address this evidence gap, the present trial was designed to evaluate the efficacy and safety of melatonin-SR capsules (2 mg) in healthy adults with poor sleep quality using both objective and subjective sleep assessments, along with quality of life (QoL) evaluation, to help in comprehensively evaluating its clinical benefits.

## 2. Results

**Figure 1:**
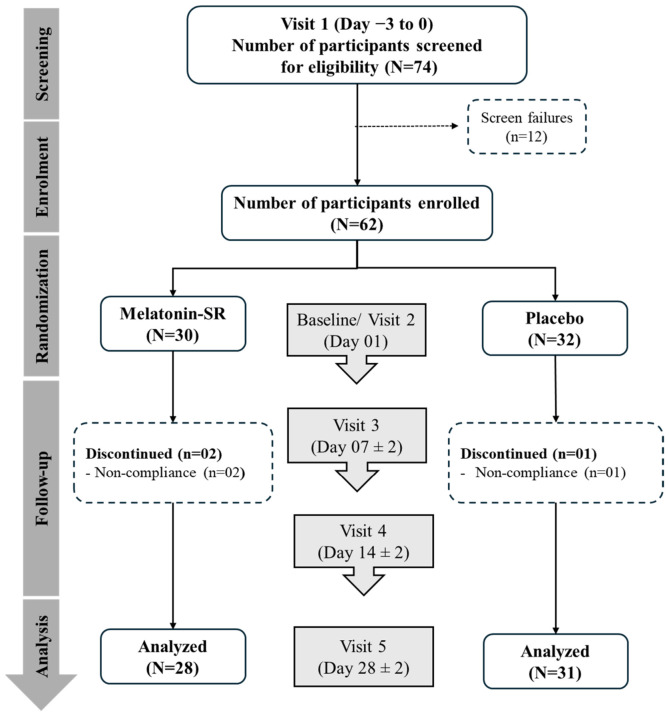
CONSORT flow diagram. Abbreviations: SR, sustained-release.

Out of 74 screened participants, 62 were enrolled and randomized to melatonin-SR (N = 30) and placebo (N = 32) groups. Of these, three participants were discontinued from the study due to non-compliance with the study protocol. As a result, a total of 59 participants completed the study and were considered for the final per-protocol analysis (melatonin-SR, *n* = 28; placebo, *n* = 31) (Figure 1).

Demographic details of the study participants at baseline are summarized in Table 1. At baseline, the mean values for demographic characteristics and each endpoint were comparable between study groups (*p* > 0.05), indicating that the melatonin-SR and placebo groups were comparable at the start of the study.

### 2.1. Change in Sleep Study Parameters Assessed Using PSG

Objective assessment of sleep quality parameters was conducted using PSG, and the following parameters were evaluated.

#### 2.1.1. Sleep Efficiency Assessment

**Figure 2:**
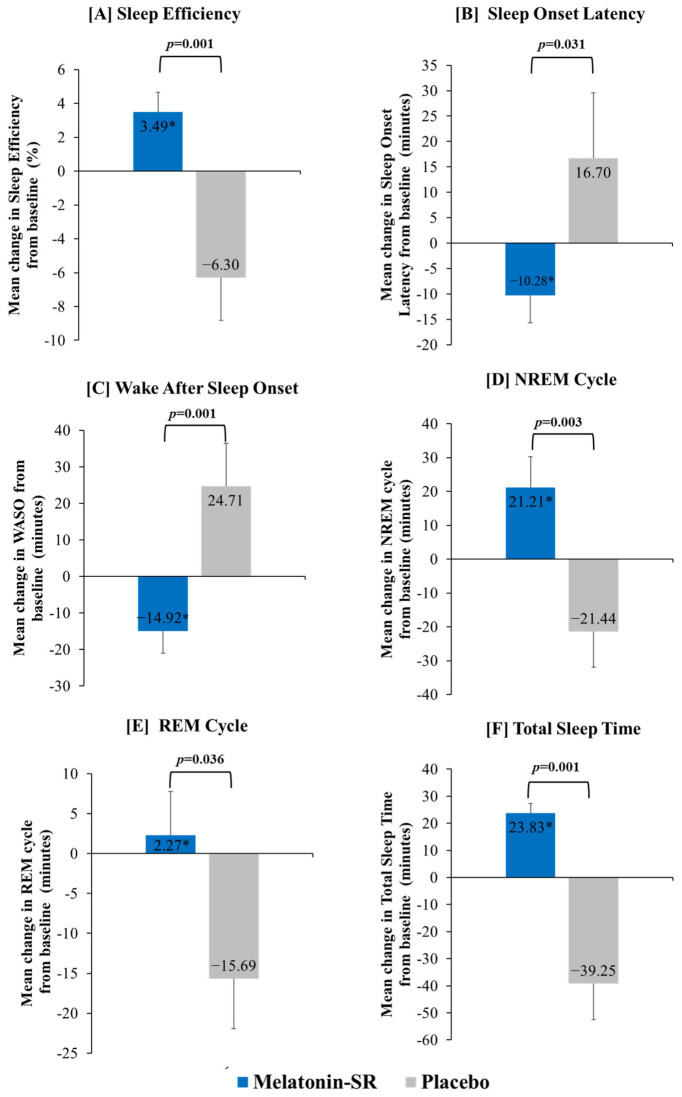
Comparison of mean change in sleep study parameters from baseline to day 28 between melatonin-SR and placebo using PSG assessment. (**A**) Sleep efficiency; (**B**) sleep onset latency; (**C**) wake after sleep onset; (**D**) NREM cycle; (**E**) REM cycle; (**F**) total sleep time. Data are presented as mean and SE (error bars represent SE). * represents statistically significant difference between groups. *p*-value derived from the Mann–Whitney U Test for between-group analysis of non-normally distributed variables; *p*-value derived from the Student’s unpaired *t*-test for between-group analysis of normally distributed variables. Abbreviations: PSG, polysomnography; REM, rapid eye movement; NREM, non-rapid eye movement; SE, standard error; SR, sustained-release; WASO, wake after sleep onset.

Participants from the melatonin-SR group reported a statistically significant increase in mean (standard error [SE]) sleep efficiency from baseline to day 28 (80.53 [2.18] vs. 84.02 [1.79]%; *p* = 0.006); while those from the placebo group reported a statistically significant decrease in sleep efficiency (77.19 [2.52] vs. 70.89 [2.20]%; *p* = 0.007). A comparative analysis of mean change from baseline to day 28 demonstrated that participants from the melatonin-SR group achieved a statistically significantly higher sleep efficiency as compared to those from the placebo group (3.49 vs. −6.30%; *p* = 0.001) (Figure 2A).

#### 2.1.2. Sleep Onset Latency (SOL) Assessment

On day 28, the mean (SE) SOL statistically significantly decreased from baseline in the melatonin-SR group (41.65 [6.62] vs. 31.37 [7.59] min; *p* = 0.041) and numerically increased in the placebo group (49.48 [8.42] vs. 66.18 [10.90] min; *p* = 0.183). A between-group comparison of mean change from baseline to day 28 demonstrated a statistically significantly greater reduction in SOL in the melatonin-SR group as compared to the mean change observed in the placebo group (−10.28 vs. 16.70 min; *p* = 0.031) (Figure 2B).

#### 2.1.3. Wake After Sleep Onset (WASO) Assessment

On day 28, the mean (SE) WASO statistically significantly decreased from baseline in the test group (90.26 [10.77] vs. 75.34 [8.74] min; *p* = 0.021) and significantly increased in the placebo group (108.30 [12.14] vs. 133.00 [10.33] min; *p* = 0.020). The comparison of mean change values from baseline to day 28 between the groups revealed a statistically significantly greater reduction in WASO duration in the melatonin-SR group compared to that observed in the placebo group (−14.92 vs. 24.71 min; *p* = 0.001) (Figure 2C).

#### 2.1.4. Non-Rapid Eye Movement (NREM) and Rapid Eye Movement (REM) Cycle Assessment

The mean (SE) NREM sleep duration statistically significantly increased on day 28 from baseline in the melatonin-SR group (NREM: 285.15 [9.56] vs. 306.35 [7.49] min; *p* = 0.025) while it decreased in the placebo group (NREM: 288.71 [8.40] vs. 267.27 [9.95] min; *p* = 0.049). The mean (SE) REM sleep duration numerically increased on day 28 from baseline in the test group (80.36 [6.02] vs. 82.63 [4.91] min; *p* = 0.684) and significantly decreased in the placebo group (79.30 [6.29] vs. 63.61 [5.53] min; *p* = 0.018). A between-group comparison demonstrated that the mean change from baseline to day 28 for both NREM and REM sleep duration in the test group was statistically significantly higher than that observed in the placebo group (NREM: 21.21 vs. −21.44 min; *p* = 0.003 and REM: 2.27 vs. −15.69 min; *p* = 0.036) (Figure 2D,E).

#### 2.1.5. Total Sleep Time (TST) Assessment

In participants from the melatonin-SR group, the mean TST (SE) statistically significantly increased from baseline to day 28 (365.50 [12.34] vs. 389.33 [9.19] min; *p* = 0.001); however, in the placebo group, it significantly decreased on day 28 (364.54 [11.85] vs. 325.29 [11.67] min; *p* = 0001). The mean change from baseline to day 28 in the melatonin-SR group was statistically significantly higher than that observed in the placebo group (23.83 vs. −39.25 min; *p* = 0.001) (Figure 2F).

### 2.2. Change in Sleep Quality Evaluated Using Pittsburgh Sleep Quality Index (PSQI)

**Figure 3:**
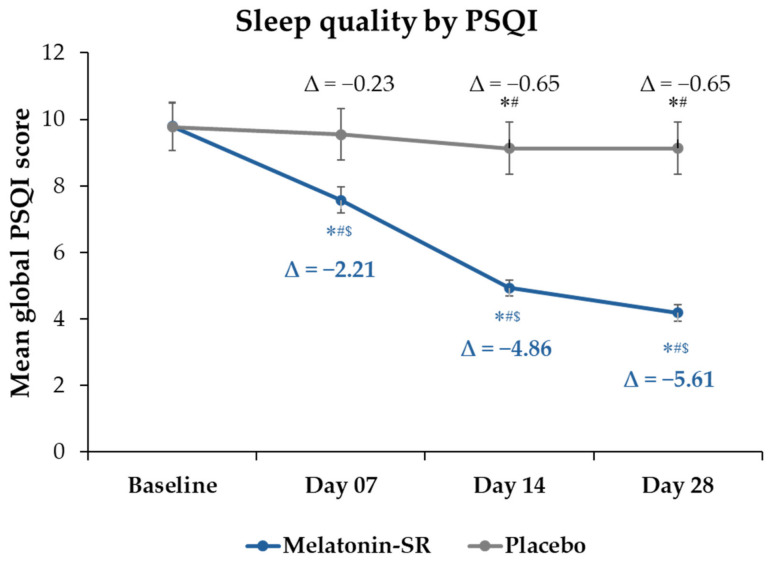
Comparison of mean change in PSQI score from baseline to day 28 between melatonin-SR and placebo. Data are presented as mean and SE (error bars represent SE). ∆ represents the mean change from baseline to the follow-up timepoints (days 07, 14, and 28). * represents *p*-value < 0.05, which indicates a statistically significant difference. # shows a comparison of mean scores between baseline and each follow-up timepoint (*p*-value derived from the Wilcoxon Signed-Rank test). $ shows comparison of the mean change from baseline to all follow-up timepoints between melatonin-SR and placebo (*p*-value derived from Mann–Whitney U test). Abbreviations: PSQI, Pittsburgh Sleep Quality Index; SE, standard error; SR, sustained-release.

Subjective assessment of sleep quality demonstrated a decreasing trend in the global PSQI scores from baseline to the end of the study duration (day 28) in both study groups. However, compared to the placebo group, participants from the test group demonstrated a statistically significantly higher reduction in PSQI global score from as early as day 07 and sustained it until the end of the study period, i.e., day 28 (mean change from baseline at day 07: −2.21 vs. −0.23, *p* = 0.001; day 14: −4.86 vs. −0.65, *p* = 0.001; and day 28: −5.61 vs. −0.65, *p* = 0.001) (Figure 3).

### 2.3. Change in QoL Assessed Using World Health Organization Five-Item (WHO-5) Well-Being Index

**Figure 4:**
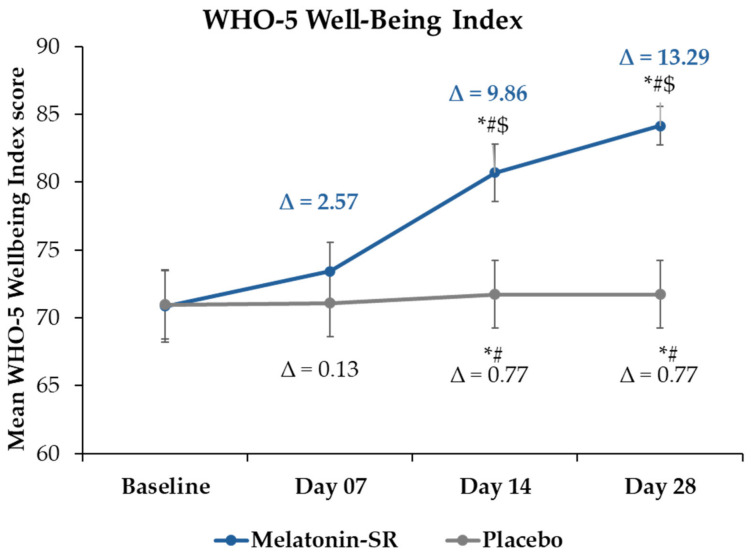
Comparison of mean change in WHO-5 Well-Being Index score from baseline to day 28 between melatonin-SR and placebo. Data are presented as mean and SE (error bars represent SE). ∆ represents the mean change from baseline to the follow-up timepoints (days 07, 14, and 28). * represents *p*-value < 0.05, which indicates a statistically significant difference. # shows comparison of mean scores between baseline and each follow-up timepoint (*p*-value derived from the Wilcoxon Signed-Rank test). $ shows comparison of the mean change from baseline to all follow-up timepoints between melatonin-SR and placebo (*p*-value derived from Mann–Whitney U test). Abbreviations: SE, standard error; SR, sustained-release; WHO, World Health Organization.

Participants in the melatonin-SR group reported statistically significant improvement in mean WHO-5 Well-Being Index score across the study period when compared with baseline (day 07: *p* = 0.017; day 14 and 28: *p* = 0.001); however, those from the placebo group reported a numerical increase from baseline at day 07 (*p* = 0.317) and a significant increase at day 14 (*p* = 0.014), with no further change at day 28. Between-group comparisons of mean change from baseline showed a numerical increase in the WHO-5 Well-Being Index score in the test group at day 07 (2.57 vs. 0.13; *p* = 0.133). Compared to the placebo group, a statistically significant improvement was observed in the test group from day 14 onward (9.86 vs. 0.77; *p* = 0.001) and was sustained until the end of the study period (day 28: 13.29 vs. 0.77; *p* = 0.001) (Figure 4).

### 2.4. Change in Sleep-Related Parameters Assessed Using Participants’ Sleep Diaries

Subjective assessment of different sleep-related parameters was performed by analyzing sleep diary data of each participant and is summarized in Table 2. A comparison of mean change values between the study groups revealed a statistically significant reduction in the duration of daytime naps, daytime fatigue levels, lights-out time, time taken to fall asleep after lights out, duration of staying awake at night, and midnight awakenings starting from day 07 onwards and a significant reduction in daytime stress levels from day 14 until the end of the study (day 28) in the melatonin-SR group compared to placebo (*p* < 0.05). Additionally, in the melatonin-SR group, a statistically significant improvement in sleep duration and rest score was observed from day 07 and sustained until day 28 as compared to the placebo group (*p* < 0.05). Also, a significant reduction in daytime sleepiness was observed in the melatonin-SR group, as reflected in the significantly lower proportion of participants taking daytime naps compared with placebo, starting as early as day 07 (73.07% vs. 100%; *p* = 0.014), which persisted through day 14 (69.23% vs. 100%; *p* = 0.007) and day 28 (23.07% vs. 96.15%; *p* = 0.001). Additionally, a significantly higher proportion of participants in the test group reported being alert during the daytime from day 14 onward (day 14: 100% vs. 70.96%; *p* = 0.001; day 28: 100% vs. 54.83%; *p* = 0.001) compared with placebo.

### 2.5. Safety Assessment

Clinical safety assessment showed no clinically significant changes in systolic or diastolic blood pressure, pulse rate, body temperature, oxygen saturation (SpO_2_), respiratory rate, or findings from physical examinations and laboratory investigations at the end of the study compared with baseline in either of the study groups. During the study, no serious adverse events (AEs) were reported. In the melatonin-SR group, a total of five participants reported nine AEs, which included headache (*n* = 04) and dizziness (*n* = 05). In the placebo group, six participants reported a total of eight AEs, including headache (*n* = 04) and dizziness (*n* = 04). All AEs were assessed as mild in intensity and deemed unlikely to be related to the IP based on causality assessment. All AEs resolved without sequelae. None of the participants discontinued the study due to AEs and both interventions were well-tolerated by participants throughout the study.

## 3. Discussion

Based on findings from a prior pharmacokinetic study demonstrating prolonged plasma melatonin exposure with melatonin-SR formulation, the present study evaluated the clinical efficacy and safety of melatonin-SR at a dose of 2 mg once at night in improving sleep parameters among healthy adults with poor sleep quality using both objective PSG-derived outcomes and validated subjective assessment tools. The key observations collectively indicate that daily administration of a single dose of melatonin-SR 2 mg for 28 days was associated with clinically relevant improvements in sleep health among this study population.

Polysomnography is the most comprehensive and objective method for assessing sleep architecture and related physiological parameters, allowing detailed characterization of sleep continuity, staging, and disturbances [21]. In the present study, PSG assessments demonstrated that melatonin-SR supplementation in a single daily dose for 28 days resulted in significant improvements in multiple objective sleep parameters compared with placebo, indicating enhanced sleep efficiency, continuity, and architecture. Sleep efficiency improved by 4.33% from baseline in the melatonin-SR group, suggesting it was effective in achieving restorative sleep, where a greater proportion of time was spent asleep while in bed. Furthermore, analysis of the magnitude of the observed treatment effect between groups using Cohen’s *d* yielded an effect size of 0.9, indicating a large treatment effect of melatonin-SR supplementation compared with placebo for sleep efficiency. This was accompanied by a significant increase (6.52%) in TST from baseline to day 28, corresponding to approximately 6 h 30 min of sleep, indicating sustained sleep throughout the night. Furthermore, improvements in sleep architecture were confirmed by significant increases in both NREM (7.44%) and REM (2.82%) sleep durations, reflecting the preservation of physiologically restorative sleep stages. According to the National Sleep Foundation guidelines, adults are recommended to achieve at least 7 h of TST and maintain a sleep efficiency of ≥85% to support optimal health and daytime functioning [22,23]. After 28 days of supplementation, participants receiving melatonin-SR achieved sleep efficiency exceeding 84% and a TST of approximately 6.5 h, values approaching the abovementioned recommended thresholds. In contrast, participants in the placebo group experienced a decline in sleep efficiency (−6.30%), resulting in a mean sleep efficiency of approximately 71% at day 28. This decrease in sleep efficiency may plausibly reflect the actual absence of effective treatment in the placebo group, and underline the efficacy of Melatonin-SR. The improvement in sleep efficiency observed with melatonin-SR (Cohen’s *d* = 0.9) reflects better sleep consolidation and maintenance in individuals experiencing poor sleep quality and can be considered clinically relevant. Collectively, these findings suggest that melatonin-SR may contribute to meaningful improvements in objective sleep outcomes in adults with poor sleep quality.

Furthermore, PSG assessment in this study revealed that melatonin-SR supplementation significantly reduced SOL (−24.68%) and WASO (−16.53%), whereas increases in these parameters were observed in the placebo group. These observations indicate a faster sleep initiation, reduced nocturnal awakenings and less fragmented sleep with this sustained-release formulation, ensuring uninterrupted sleep throughout the night. Collectively, these PSG-derived observations suggest that melatonin-SR confers multidimensional benefits on sleep initiation and maintenance. The observed improvements are consistent with the earlier evaluated pharmacokinetic profile of the test product, which demonstrated prolonged nocturnal melatonin exposure that more closely mimics endogenous melatonin secretion patterns, thereby supporting continuous sleep throughout the night [17]. Importantly, the use of objective PSG assessments strengthens the clinical relevance of the pharmacokinetic study findings.

These results are broadly consistent with prior PSG-based investigations of prolonged-release melatonin. In a study of elderly patients (≥55 years) with primary insomnia, the administration of 2 mg prolonged-release melatonin for three weeks resulted in a significant reduction in SOL by 9 min compared with placebo (*p* = 0.02), supporting its role in facilitating sleep initiation. However, improvements in sleep continuity and sleep architecture were not observed in this population [19]. A more recent study conducted in 24 healthy older adults (>55 years) without sleep complaints demonstrated that 5 mg melatonin significantly increased sleep efficiency, TST, and sleep duration during both biological day and night, primarily through increases in stage 2 NREM sleep and modest reductions in awakenings [20]. On the contrary, a randomized double-blind placebo-controlled study by Almeida Montes LG et al. evaluating a low-dose sustained-release melatonin formulation (1 mg) in participants with primary insomnia reported no significant differences in sleep EEG parameters, sleep duration, subjective sleep quality, or adverse effects compared with placebo [24]. Similarly, another clinical trial in healthy middle-aged adults (55–64 years) reported that prolonged-release melatonin did not significantly affect PSG sleep measures compared with placebo, suggesting that dose, population characteristics, and formulation design are crucial factors influencing clinical outcomes [25]. Taken together, these findings, along with the results of the present study, suggest that sustained-release melatonin formulations administered within the 2–5 mg dose range may contribute to improvements in objective sleep parameters.

In addition to objective PSG assessments, subjective sleep quality was evaluated using the validated tool, PSQI, to determine whether participant-reported perceptions of sleep corresponded with physiological sleep improvements. Evaluation of the PSQI global scores demonstrated a progressive and clinically meaningful improvement in subjective sleep quality in the melatonin-SR group over the 28-day study period. A significant reduction from baseline was observed as early as day 07 (% change from baseline: −22.57%), which further decreased at day 14 (−49.64%) and day 28 (−57.30%), compared with the placebo group. These sustained improvements in perceived sleep quality align with the objective PSG findings, indicating concordance between subjective assessments and physiological sleep parameters. Together, these observations further support the clinical efficacy of melatonin-SR in improving overall sleep quality among healthy individuals with poor sleep quality. Previous randomized controlled trials and meta-analyses have consistently shown that melatonin supplementation improves subjective sleep quality as measured by PSQI; however, the magnitude of benefit has generally been modest and often poorly aligned with objective sleep measures. A meta-analysis of 23 randomized clinical trials reported an average PSQI reduction of approximately 1.2 points (95% CI: −1.77, −0.71, *p* = 0.000) with melatonin doses ranging from 2 to 10 mg across heterogeneous populations, including individuals with sleep disorders (mean reduction: −0.67; 95% CI −0.98, −0.37, *p* = 0.000) and metabolic disorders (mean reduction: −2.74; 95% CI: −3.48, −2.00; *p* = 0.000) [9]. Similarly, in elderly individuals (>65 years) with primary insomnia, prolonged-release melatonin (2 mg) resulted in relatively small but statistically significant PSQI reductions at both 2 weeks (mean reduction: −0.64; 95% CI: −1.25, −0.02; *p* = 0.042) and 26 weeks (mean reduction: −0.70; 95%CI: −1.17, −0.23; *p* = 0.003) [26]. Furthermore, larger reductions have been reported in certain clinical settings, such as hospitalized or critically ill populations [27], although these findings may not be directly comparable due to differences in study design and trial population. Notably, the magnitude and rapid onset of PSQI improvement observed in the present study (reduction by 4.86 points on day 14 and 5.61 points on day 28) exceed the pooled average effects observed across different populations. This pronounced response in healthy individuals with poor sleep quality suggests that sustained-release melatonin may confer greater sleep benefits when administered early in the trajectory of sleep disturbance, particularly in otherwise healthy individuals with sleep disturbances, due to consistent plasma melatonin levels compensating for disturbed endogenous melatonin secretion, resulting in undisturbed sleep.

Poor sleep quality is well recognized to be associated with impairments in QoL, with individuals experiencing disturbed sleep consistently reporting lower well-being compared to individuals with good sleep quality [28]. Therefore, in the present study, QoL was assessed using the WHO-5 Well-Being Index in healthy individuals with poor quality of sleep to evaluate the broader impact of melatonin-SR supplementation beyond sleep parameters. Participants receiving melatonin-SR demonstrated a positive trend of improvement in overall WHO-5 well-being scores across the study duration, with a statistically significant improvement in subjective psychological well-being from day 14 onward (13.91%), sustained through day 28 (18.76%), compared with the placebo group. These findings suggest that improvements in sleep quality with melatonin-SR may translate into meaningful benefits in overall QoL. Consistent with these observations, prior studies evaluating prolonged-release melatonin in individuals with insomnia have reported improvements in QoL measures, supporting a potential role for sustained-release melatonin formulations in enhancing sleep-related QoL outcomes [26,29].

Lastly, sleep diary assessments provided additional subjective evidence supporting the benefits of melatonin-SR supplementation across multiple sleep-related domains. Compared with placebo, participants in the melatonin-SR group demonstrated significant reductions in daytime napping, daytime fatigue, daytime sleepiness, and nocturnal awakenings from day 07 onward, along with a reduction in daytime stress and improvement in daytime alertness from day 14. These improvements were accompanied by significant increases in sleep duration and restfulness scores from day 07 through the end of treatment, further reinforcing the consistency of subjective sleep improvements observed with melatonin-SR. Collectively, these observations indicate enhanced perceived sleep continuity, nighttime restfulness, and daytime alertness in the melatonin-SR group, reinforcing the objective improvements observed in PSG outcomes. These observations align with the published literature wherein sleep diary assessment was used to capture both nocturnal sleep quality and daytime functioning following prolonged-release melatonin supplementation [29,30]. A postmarketing surveillance study also reported patient-reported effectiveness of prolonged-released melatonin in improving sleep quality and morning alertness among elderly patients with insomnia [31].

Safety evaluations indicated that melatonin-SR exhibited a favorable tolerability profile, with no clinically meaningful differences observed between the melatonin-SR and placebo groups across assessed safety parameters. All reported adverse events were mild in intensity, transient in nature, and considered unlikely to be related to the IP, with complete resolution and no sequelae. These findings suggest that once-daily administration of 2 mg melatonin-SR is generally well tolerated in healthy adults with poor sleep quality. The observed safety profile is consistent with previously published clinical trials, further supporting its suitability for use as a nutraceutical intervention for sleep health [19,29,30,32,33].

To our knowledge, evidence evaluating sustained-release melatonin supplementation in healthy adults with poor sleep quality remains limited, and the present randomized, placebo-controlled trial contributes novel clinical data in this population. The study demonstrated the efficacy of a low-dose (2 mg) melatonin-SR supplement in improving sleep health, thereby adding meaningful evidence to the existing literature on nutraceutical-based sleep interventions. Furthermore, the inclusion of PSG, a comprehensive and objective method for assessing sleep architecture and related physiological parameters, in the present study enhanced its methodological rigor by allowing reliable quantification of treatment-related changes in sleep outcomes and by substantiating subjective findings with objective PSG outcomes. Moreover, conducting PSG in a home-based setting facilitated the collection of sleep data in the real-world environment with habitual sleeping conditions at home, thereby reducing sleep disruption caused by unfamiliar laboratory conditions and potentially mitigating first-night effects. The use of multiple validated subjective assessment tools further allowed a holistic evaluation of sleep quality and daytime well-being. It is noteworthy that several objective sleep parameters deteriorated in the placebo group over 28 days, which may reflect regression of baseline sleep disturbances. The reasons underlying this deterioration are not entirely clear but factors such as natural variability in sleep patterns, progression of baseline sleep disturbances, or the absence of active sleep-supportive intervention during the study period may have contributed. However, melatonin-SR consistently mitigated this decline and produced net improvements across sleep parameters. Nevertheless, the study duration was limited to 28 days, which was appropriate for assessing short-term efficacy and tolerability; however, designing a future trial with a longer duration will further help in the evaluation of sustained benefits with continued use. Interestingly, this design strengthens the relevance of the findings to a broader, real-world population seeking non-pharmacological sleep support. Future studies with extended follow-up periods and the inclusion of diverse sleep phenotypes would further elucidate the long-term clinical utility and generalizability of sustained-release melatonin supplementation.

## 4. Materials and Methods

### 4.1. Study Design and Ethical Considerations

This was a multicenter, randomized, double-blind, parallel-group, prospective placebo-controlled clinical trial. The trial was conducted at two study sites (Kulkarni Clinic, Kasturba Housing Society, Pune, Maharashtra, India and Dhanwantari Hospital, Rasta Peth, Pune, Maharashtra, India) between 29 April 2025 and 4 September 2025.

Ethics committees at both study sites approved the study protocol (Central Independent Ethics Committee, Maharashtra, India [Registration number: ECR/390/Indt/MH/2024; Approval Date: 9 April 2025] and Jivanrekha Ethics Committee, Maharashtra, India [Registration number: ECR/1580/Inst/MH/2021; Approval Date: 4 May 2025]). The study protocol adhered to the ethical guidelines set forth by the Declaration of Helsinki for research involving human participants. The study was conducted in compliance with the Indian Council of Medical Research (ICMR) guidelines—National Ethical Guidelines for Biomedical and Health Research Involving Human Participants, 2017; International Conference on Harmonization-Good Clinical Practices (ICH-GCP) guidelines E6 (R3); NDCT RULES 2019; and the Declaration of Helsinki (Brazil, October 2013). This study was registered with the Clinical Trials Registry-India (CTRI) on 17 April 2025 (CTRI Number: CTRI/2025/04/085087). Each study participant provided written informed consent before initiation of the study.

### 4.2. Study Population

Participants were recruited through site-based screening at the study clinics. Individuals presenting to the clinics with sleep-related complaints, including sleep disturbances, as part of routine healthcare consultations were informed about the study by the principal investigator (PI). Potentially interested individuals were screened according to predefined eligibility criteria, and those who met the inclusion criteria and provided written informed consent were enrolled in the study.

#### Eligibility Criteria

Healthy adult male and non-pregnant female participants aged between 30 and 60 years, with a body mass index (BMI) ranging from 18.5 to 29.9 kg/m^2^, who reported poor sleep quality (determined by a PSQI global score >5 at the screening visit) and experienced at least three episodes of sleep disturbances in the preceding month, were enrolled for the study. Additional inclusion criteria comprised participants who were willing to abstain from any digital activity for at least three hours prior to undergoing PSG analysis, and maintain their usual dietary habits, physical activity levels, and stable body weight throughout the study duration, avoiding any significant lifestyle modifications. Female participants of childbearing potential were required to use a medically acceptable form of contraception during the study. Women participants who were amenorrheic for at least one year or had undergone hysterectomy and/or bilateral oophorectomy were considered non-childbearing. All laboratory test results and screening assessments of eligible participants were required to be within normal limits or deemed not clinically significant by the PI. Lastly, participants who demonstrated willingness to provide written informed consent and to comply with all study procedures, including PSG evaluation, blood sampling, routine urine analysis, and urine pregnancy testing (for women of childbearing potential), both before and after supplementation, were included in the study.

The participants were excluded from the study if they had lifestyles that could interfere with sleep patterns, such as shift work or frequent travel across time zones, causing jet lag. Other exclusion criteria were participants with history of any sleep disorders related to psychiatric disorders (for example, depression, anxiety, dementia) or secondary medical conditions (such as sleep apnea, circadian rhythm sleep disorder) or any chronic medical condition that was likely to be the cause of the sleep problem; pregnant women, breastfeeding women, women planning pregnancy, or post-menopausal women using hormone replacement therapy; history of known allergy to investigational products (IP); history or presence of any clinically significant uncontrolled systemic disorder, including, but not limited to, cardiovascular, gastrointestinal, endocrinologic, hematologic, hepatic, immunologic, urologic, pulmonary, dermatologic, renal and/or other major diseases; history or presence of alcohol intake (>2 standard drinks/day) or the use of recreational drugs or addiction to nicotine; and excess consumption of tea (≥500 mL/day), coffee (≥400 mL/day), or energy drinks (≥250 mL/day). Participants were also excluded if they were currently using any prescription medicine, herbal supplements, any over-the-counter product or multivitamins for sleep or anxiety or any other psychological condition, or any other prescription product that has a known side effect of causing somnolence or sleep problems within one month prior to the screening visit. Individuals who had participated in other clinical trials involving investigational or marketed products within three months prior to screening, or those considered not eligible for participation by the PI, were also excluded.

### 4.3. Study Products

The test product was Melatonin-SR capsules containing melatonin SR granules 4.44 mg equivalent to 2 mg melatonin (Melotime^™^). Placebo capsules contained only inactive substances and were manufactured as identical capsules that matched the color, size, weight, and shape of the test product. Both test and placebo products were manufactured by Nutriventia Private Limited, Mumbai, India.

### 4.4. Randomization and Blinding

After confirming eligibility, each participant was assigned a unique randomization number according to the predefined randomization schedule. The randomization schedule was generated by an independent statistician using a computer-generated simple randomization method. Participants were randomized in a 1:1 ratio to receive either melatonin-SR capsules or placebo capsules. The study participants, sponsor, investigators, and all site personnel involved in study conduct, assessments, data entry, and data evaluation were blinded to the treatment assignments until database lock. Blinding was implemented by labeling the IP through an assigned unblinded individual. At the study site, sealed envelopes containing randomization codes and blinded treatment allocation details were securely stored under controlled access in the custody of the PI. Emergency unblinding was permitted only in cases of medical or surgical emergencies where knowledge of the treatment assignment was essential for appropriate clinical management.

### 4.5. Study Procedure

**Figure 5:**
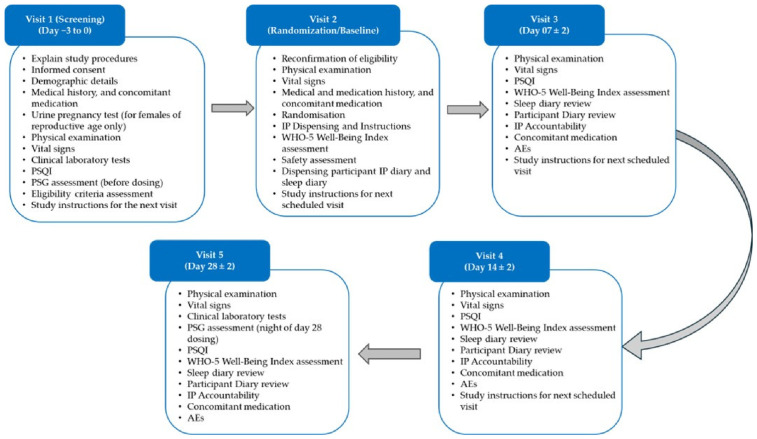
Study procedure flow-chart. Abbreviations: AEs, adverse events; IP, investigational product; PSG, polysomnography; PSQI, Pittsburgh Sleep Quality Index; WHO, World Health Organization.

The study was conducted for approximately 34 days. This included 4 days of screening (Visit 1 [day −3 to 0]), 28 days of treatment (Visit 2 [baseline/day 01), Visit 3 [day 07 ± 2], Visit 4 [day 14 ± 2], and Visit 5 [day 28 ± 2]) (Figure 5). During the screening visit, all participants provided written informed consent. Screening evaluations, including medical history, physical examinations, demographic and anthropometric measurements, vital signs measurement, urine pregnancy test (for women in the reproductive age group), clinical laboratory examinations (including hematology, biochemistry and urine analysis) and PSQI assessment, were conducted in all study participants. During the baseline visit (day 01), medical and medication history, physical examination, vital signs assessment and reconfirmation of eligibility criteria were conducted, and all eligible participants were randomized to receive either 2 mg melatonin-SR or placebo capsules. The IP containers containing 34 capsules of either melatonin-SR or placebo were dispensed to each participant. Participants were instructed to take one capsule orally with water once daily at night 0.5–1.0 h before sleep (preferably between 09:00 and 09:30 PM) for 28 days. This dosing window was selected to standardize the supplement administration relative to the scheduled PSG recording timings and planned lights-off time (22:30), thereby minimizing variability in dosing timings across participants. A home-based PSG was conducted on the night of the screening visit (prior to baseline visit) and on day 28 after dosing by a qualified and certified sleep technician. In accordance with the risk-mitigation plan, to minimize the study dropout rates, the evaluation during the screening visit was considered as the baseline PSG assessment. Quality of life was assessed using the WHO-5 Well-Being Index at baseline visit and days 07, 14, and 28. For the day 07 assessment, the WHO-5 questionnaire was administered with an adjusted recall period to reflect the evaluation on day 07. Sleep quality assessment was repeated on days 07, 14 and 28 using PSQI. Additionally, all individuals were given a participant diary, which included a sleep record section where participants documented their pre- and post-sleep-related activities and daily sleep patterns and an IP intake and AEs record section where they recorded details of IP administration, any AEs experienced, and any concomitant medications taken throughout the study period. They were advised to fill these diaries on a daily basis. These diaries were reviewed by the study team at each visit for sleep-related parameters, IP compliance and safety assessment. Unused IPs were collected from participants on day 28. Treatment compliance was considered adequate if participants consumed, on average, at least 80% of the scheduled IP doses. Assessment of physical examinations, vital signs, concomitant medication use and AE monitoring were conducted at all study visits, while laboratory clinical assessments were conducted at screening (visit 1) and at the end of the study (day 28).

During the entire study period, participants were instructed to refrain from taking any medications or supplements known to interfere with sleep or cause somnolence at the time of enrollment, other than the IP. The use of any concomitant medications not affecting the parameters of the study was allowed at the discretion of the PI. In the event of illness requiring additional medication, participants were required to notify the PI or designated study personnel immediately.

### 4.6. Endpoints

The primary efficacy endpoint was to evaluate and compare the mean change in sleep efficiency measured by PSG from baseline to the end of the treatment (day 28) between the melatonin-SR and placebo groups. The secondary endpoints included the following: (i) to compare mean change from baseline to day 28 between the two study groups in PSG-derived sleep parameters including SOL, WASO, the duration of NREM sleep stages and REM sleep stage, and TST; and (ii) to compare the mean change in participant-reported subjective assessments from baseline to interim follow-up visits (i.e., day 07 and 14) until the end of the study (day 28) between the melatonin-SR and placebo groups. These included PSQI global score, WHO-5 Well-Being Index, and sleep-related parameters recorded in the participant’s sleep diary. Additionally, safety assessments were conducted, which included an evaluation of the incidence, frequency, and severity of AEs and treatment-emergent adverse events (TEAEs) throughout the study duration.

### 4.7. Study Assessment Tools

#### 4.7.1. Polysomnography

Polysomnography, the gold-standard diagnostic tool for evaluating sleep-related disorders, was employed in this study to comprehensively assess sleep architecture and identify physiological changes during sleep. This technique utilizes multiple components, each designed to measure specific physiological functions, which subsequently aid in evaluating underlying causes of sleep disturbances [18,21,34].

Overnight sleep assessments were conducted using a validated and calibrated home-based portable SOMNOtouch RESP PSG device (manufactured by SOMNOmedics GmbH, Randersacker, Germany). This device includes a headbox with an electroencephalogram (EEG), which measures brain activity and helps classify sleep stages (NREM and REM sleep); an electrooculogram (EOG) which monitors eye movements, crucial for identifying REM sleep; an electromyogram (EMG), which records muscle activity, particularly in the chin (to assess REM sleep atonia) and limbs (to detect movement disorders); a combination sensor that captures periodic limb movements; an electrocardiogram (ECG), which monitors heart rate and rhythm during sleep and respiratory efforts (chest/abdominal effort belts) by detecting movement of the chest and abdomen; and 11 internal channels that measure blood oxygen saturation (SpO_2_), pulse rate, and plethysmogram. Previous studies have demonstrated that home-based PSG reduces first-night effects by minimizing sleep disruptions caused by an unfamiliar environment and provides reliable sleep architecture data comparable to laboratory-based PSG assessment [35,36,37,38].

A trained sleep technician visited each participant’s residence to apply all required leads and sensors and provided instructions for their removal the following morning upon awakening. Participants were instructed to maintain their usual daytime schedule, abstain from digital device use for three hours before the assessment, avoid napping on the day of the assessment, and refrain from alcohol, caffeine (including coffee, tea, cocoa, and chocolate), sedatives, and stimulants for 24 h prior to the recording. Participants were advised to consume their regular meals, including dinner, to wear comfortable sleep attire, and they were permitted to keep reading material with them before sleep onset. The sleep parameters evaluated included sleep efficiency (defined as the percentage of total time in bed actually spent asleep [Sleep efficiency = total sleep time/time in bed × 100]), SOL (defined as the duration of time in minutes between lights turned off as the participant attempts to sleep and the first sleep epoch), WASO (defined as total periods of wakefulness in minutes occurring after sleep onset until the final awakening), REM and NREM sleep cycle, and TST (defined as total amount of sleep time from sleep onset to sleep offset). All PSG recordings were scored in accordance with the AASM Manual for the Scoring of Sleep and Associated Events. The total time required to complete the PSG test was approximately 8 h (lights-off and the beginning of the PSG recordings were set at 22:30; lights-on and the termination of the PSG recordings were set at 6:30). All recorded PSG sleep data were reviewed and analyzed by the designated sleep specialist or PI to determine sleep architecture and identify sleep disturbances and overall sleep quality.

#### 4.7.2. Pittsburgh Sleep Quality Index

The PSQI is a widely used, validated tool for assessing overall sleep quality and sleep-related behaviors. It involves the assessment of seven components, which include subjective sleep quality, sleep latency, sleep duration, habitual sleep efficiency, sleep disturbances, use of sleep medications, and daytime dysfunction. Each component is scored on a 0–3 Likert scale, where a score of 0 reflects no difficulty and a score of 3 represents severe difficulty. The component scores are summed to generate a global PSQI score, which ranges from 0 to 21. A global score > 5 is considered indicative of poor sleep quality relative to clinical and laboratory measures [38].

#### 4.7.3. WHO-5 Well-Being Index

The WHO-5 Well-Being Index is a brief, validated questionnaire designed to assess subjective psychological well-being. It consists of five positively worded items that capture how individuals have felt over the preceding two weeks. Each item is rated on a 6-point scale ranging from 0 (“at no time”) to 5 (“all of the time”), yielding a raw total score between 0 and 25, where higher scores reflect greater well-being. As scales measuring health-related quality of life are conventionally translated to a percentage scale from 0 (absent) to 100 (maximal), it is recommended to multiply the raw score by 4. A percentage score below 50 (or a raw score below 13) has been suggested as a cut-off for poor mental well-being, and a score of 100 represents the best possible mental well-being [22,39,40].

#### 4.7.4. Sleep Diary Assessment

Participants were instructed to complete a daily sleep diary from baseline to day 28 to record both pre-sleep and post-sleep parameters. Sleep diary data were evaluated at baseline and on days 07, 14, and 28. Participants recorded pre-sleep parameters each night before going to sleep, and these included the number and duration of daytime naps, the number of participants feeling sleepy during the day, daytime fatigue level, and daytime stress level. Post-sleep parameters were recorded each morning after awakening. These included lights-off time, time taken to fall asleep after lights out, number of times participants woke up during the night, duration of staying awake during the night, number of hours of sleep during night, time of waking up in the morning, and rest score.

### 4.8. Statistical Analysis

The sample size calculation was done using Statistical Package for Social Sciences (SPSS), version 10.0. Based on the previous study results, assuming a margin of error of 1.9 min, a power of 80%, an effect size of 0.9, and a type one error rate (alpha) of 5%, the number of participants required per group to find an effect of melatonin on sleep parameters evaluated by PSG was estimated as 56 [18]. Considering a 10% dropout rate, we aimed to recruit a total of 60 participants (30 participants per group) for this study.

Data were analyzed using SPSS software, version 30.0 (SPSS Inc., Chicago, IL, USA). The data of all participants who completed all the study visits were considered for per-protocol study analysis. All efficacy evaluations were conducted in the per-protocol population and safety evaluations were conducted in the intention-to-treat population. The normality of data was assessed using the Shapiro–Wilk test. Descriptive analysis was used to summarize continuous parameters using the mean, standard deviation (SD) or SE and categorical parameters using frequencies and percentages. The comparison of mean values between baseline and each follow-up visit within each group was performed using the Paired Samples *t*-test for normally distributed parameters and the Wilcoxon signed-rank test for non-normally distributed parameters. Comparative analysis of mean changes from baseline to each follow-up visit between the two groups was performed using an independent sample *t*-test (for normally distributed parameters) or the Mann–Whitney U test (for non-normally distributed parameters). All *p*-values were reported based on a two-sided significance test, and a *p* < 0.05 was considered statistically significant.

## 5. Conclusions

The sustained-release melatonin capsules (2 mg) demonstrated consistent efficacy across both objective and subjective sleep assessments in healthy adults with poor sleep quality. PSG assessments showed that the melatonin-SR group had significant improvement in sleep efficiency and significantly greater maintenance of NREM and REM sleep stages, with significant reductions in sleep onset latency and nocturnal awakenings as compared to the placebo group. These objective improvements were supported by participant-reported outcomes, with significant changes observed in PSQI scores, WHO-5 Well-Being Indices, and sleep diary parameters, reflecting enhanced sleep quality, sleep duration, restfulness, and daytime functioning, along with reductions in sleep fragmentation, daytime sleepiness, fatigue, and stress. Overall, these benefits resulted in improvements in the QoL of the participants in the test group. Collectively, the results suggest that 28 days of melatonin-SR supplementation may offer a well-tolerated and effective nutraceutical approach for improving sleep quality and overall well-being in adults experiencing poor sleep quality.

## Figures and Tables

**Table 1:** Demographic characteristics at baseline.

Parameters	Melatonin-SR (N = 30)	Placebo (N = 32)	Total (N = 62)	*p*-Value
Age (years)	40.63 (8.05)	39.25 (5.62)	39.92 (6.88)	0.439
Sex (*n*, %)				
Men Women	15 (50.00) 15 (50.00)	20 (62.50) 12 (37.50)	35 (56.50) 27 (43.50)	0.321
Body weight (kg)	66.02 (11.31)	66.09 (9.04)	66.05 (10.12)	0.979
Height (cm)	164.60 (10.21)	164.81 (6.93)	164.71 (8.60)	0.925
BMI (kg/m^2^)	24.11 (2.35)	24.30 (2.73)	24.20 (2.53)	0.769

Data are presented as means (SD) unless otherwise specified. The data was analyzed using Student’s *t*-test for all parameters except sex, where Fisher’s exact test was used. BMI, body mass index; SD, standard deviation; SR, sustained-release.

**Table 2:** Sleep-related parameters using sleep diary assessment.

Sleep Diary Questionnaire	Melatonin-SR				Placebo			
	Baseline	Day 07	Day 14	Day 28	Baseline	Day 07	Day 14	Day 28
Mean number of daytime naps	1.62 (0.57)	1.63 (0.60)	1.33 *^#^ (0.49)	1.00 *^#^ (0.00)	1.96 (0.66)	2.00 (0.69)	1.92 (0.74)	2.00 (0.76)
Mean change from baseline	-	−0.05	−0.33	−1.17 *^$^	-	0.04	−0.04	0.04
Mean duration of daytime naps (min)	38.08 (13.86)	37.89 *^#^ (18.43)	33.33 *^#^ (14.14)	10.00 *^#^ (0.00)	38.08 (18.77)	38.08 (18.77)	37.12 (18.88)	39.80 (20.02)
Mean change from baseline	-	−3.68 *^$^	−8.89 *^$^	−21.67 *^$^	-	0.00	−0.96	1.80
Mean daytime fatigue level	2.29 (1.08)	1.96 *^#^ (1.43)	1.36 *^#^ (1.13)	0.25 *^#^ (0.44)	2.23 (0.92)	2.32 (1.01)	2.52 (1.34)	2.65 *^#^ (1.38)
Mean change from baseline	-	−0.32 *^$^	−0.93 *^$^	−2.04 *^$^	-	0.10	0.29	0.42
Mean daytime stress level	2.29 (1.21)	2.04 *^#^ (1.48)	1.39 *^#^ (1.10)	0.25 *^#^ (0.44)	2.23 (0.80)	2.26 (0.86)	2.19 (1.01)	2.42 (1.36)
Mean change from baseline	-	−0.25	−0.89 *^$^	−2.04 *^$^	-	0.03	−0.03	0.19
Mean lights-out time (h)	22.98 (0.61)	22.84 *^#^ (0.41)	22.45 *^#^ (0.44)	21.89 *^#^ (0.34)	22.44 (0.70)	22.52 (0.69)	22.45 (0.73)	22.53 (0.46)
Mean change from baseline	-	−0.14 *^$^	−0.54 *^$^	−1.09 *^$^	-	0.08	0.01	0.09
Mean time taken to fall asleep after lights out (min)	42.50 (13.78)	33.75 *^#^ (7.77)	23.57 *^#^ (4.88)	14.46 *^#^ (3.14)	38.06 (11.38)	43.06 *^#^ (13.21)	39.03 (11.06)	41.94 *^#^ (14.47)
Mean change from baseline	-	−8.75 *^$^	−18.93 *^$^	−28.04 *^$^	-	5.00	0.97	3.87
Mean number of times participants woke up during night	1.82 (0.77)	1.14 *^#^ (0.93)	0.50 *^#^ (0.51)	0.00 *^#^ (0.00)	1.61 (0.99)	1.81 (0.79)	1.87 *^#^ (0.99)	1.94 *^#^ (1.00)
Mean change from baseline	-	−0.68 *^$^	−1.32 *^$^	−1.82 *^$^	-	0.19	0.26	0.32
Mean duration of staying awake during the night (min)	11.07 (5.83)	5.29 *^#^ (5.03)	2.75 *^#^ (3.15)	0.00 *^#^ (0.00)	10.65 (6.55)	12.90 *^#^ (5.59)	14.84 *^#^ (8.71)	15.65 *^#^ (8.92)
Mean change from baseline	-	−5.79 *^$^	−8.32 *^$^	−11.07 *^$^	-	2.26	4.19	5.00
Mean number of hours of sleep at night (h)	7.13 (0.43)	7.44 *^#^ (0.56)	8.48 *^#^ (0.62)	9.45 *^#^ (0.50)	8.13 (1.16)	8.10 (1.13)	8.18 (1.10)	8.00 *^#^ (1.05)
Mean change from baseline	-	0.31 *^$^	1.36 *^$^	2.32 *^$^	-	−0.03	0.05	−0.13
Mean time of waking up in morning (h)	6.11 (0.42)	6.28 *^#^ (0.65)	6.96 *^#^ (0.71)	7.34 *^#^ (0.51)	6.86 (0.86)	6.91 (0.88)	6.92 *^#^ (0.94)	6.81 (0.87)
Mean change from baseline	-	0.17 *^$^	0.86 *^$^	1.23 *^$^	-	0.05	0.05	−0.05
Mean rest score	2.79 (0.42)	3.75 *^#^ (0.44)	4.39 *^#^ (0.50)	5.00 *^#^ (0.00)	3.45 (0.51)	3.29 *^#^ (0.74)	3.29 *^#^ (0.74)	2.55 *^#^ (0.62)
Mean change from baseline	-	0.96 *^$^	1.61 *^$^	2.21 *^$^	-	−0.16	−0.16	−0.90

Data are presented as means (SD). * represents *p*-value < 0.05, which indicates a statistically significant difference. ^#^ shows comparison of mean scores between baseline and each follow-up timepoint (*p*-value derived from the Wilcoxon Signed-Rank test for non-normally distributed variables or Student’s paired *t*-test for normally distributed variables). ^$^ shows comparison of the mean change from baseline to all follow-up timepoints between melatonin-SR and placebo (*p*-value derived from Mann–Whitney U test for non-normally distributed variables or Student’s unpaired *t*-test for normally distributed variables). Abbreviations: SD, standard deviation; SR, sustained-release.

## Data Availability

The data that support the findings of this study are available from the corresponding author upon reasonable request.

## Abbreviations

The following abbreviations are used in this manuscript:

AEs	Adverse event
AASM	American Academy of Sleep Medicine
BMI	Body mass index
CDC	Centers for Disease Control and Prevention
CTRI	Clinical Trials Registry-India
IP	Investigational product
ICMR	Indian Council of Medical Research
ICH-GCP	International Conference on Harmonization-Good Clinical Practices
Melatonin-SR	Sustained-release melatonin formulation
NREM	Non-rapid eye movement
NHIS	National Health Interview Survey
PI	Principal investigator
PSG	Polysomnography
PSQI	Pittsburgh Sleep Quality Index
QoL	Quality of life
REM	Rapid eye movement
SR	Sustained-release
SD	Standard deviation
SE	Standard error
SOL	Sleep onset latency
SpO_2_	Blood oxygen saturation
TEAEs	Treatment-emergent adverse events
TST	Total sleep time
WASO	wake after sleep onset
WHO	World Health Organization
